# Synchronous surgery and immediate reconstruction of double primary cancers of the ovary and breast

**DOI:** 10.1097/MD.0000000000029607

**Published:** 2022-08-12

**Authors:** Chao-Hsin Huang, Yur-Ren Kuo, Yu-Chieh Chen, Shen-Liang Shih

**Affiliations:** a Department of Post-baccalaureate Medicine, Kaohsiung Medical University, Kaohsiung, Taiwan; b Division of Plastic Surgery, Department of Surgery, Kaohsiung Medical University Hospital, Kaohsiung, Taiwan; c Department of Surgery, Faculty of Medicine, College of Medicine, Kaohsiung Medical University, Kaohsiung, Taiwan; d Department of Biological Sciences, National Sun Yat-sen University, Kaohsiung, Taiwan; e Department of Obstetrics and Gynecology, Kaohsiung Medical University Chung-Ho Memorial Hospital, Kaohsiung, Taiwan; f Division of Oncology and Surgery, Department of Surgery, Kaohsiung Medical University Chung-Ho Memorial Hospital, Kaohsiung, Taiwan; g Department of Health Business Administration, Meiho University, Pingtung, Taiwan.

**Keywords:** case report, double primary cancers, synchronous surgery

## Abstract

**Introduction:**

There has been an ongoing debate about the benefits and risks between synchronous surgery and two independent surgery of ovary tumor removal and breast reconstruction. Here we report a synchronous oncological surgery and immediate postmastectomy reconstruction of double primary cancers of the ovary and breast.

**Patient concerns:**

A 58-year-old woman presented with a right breast lump and ascites.

**Diagnosis:**

Computed tomography (CT) indicated synchronous breast and ovarian cancer with multiple metastases. Double primary mammary and ovarian cancer was confirmed after a series of evaluations, such as core needle biopsy of the breast tumor.

**Interventions and outcomes:**

Synchronous surgery and immediate reconstruction of double primary cancers of the ovary and breast were performed. Post-operative results showed complete resection of ovarian tumor, no post-operative complication, and excellent life quality.

**Conclusion:**

Synchronous surgery is warranted as a treatment option for selected cases of double primary cancer. The surgery not only achieved complete removal of one cancer and reduction of the other but also reached excellent breast reconstruction and body shape recovery.

## 1. Introduction

There has been an ongoing debate about the benefits and risks between synchronous surgery and two independent surgery of ovary tumor removal and breast reconstruction.^[1–3]^ Here we reported a unique case with naturally formed flap volume for breast reconstruction, which is the best fit for a synchronous surgery. The challenge for our patient was that two independent operations led to reciprocal but detrimental effects on each other’s surgical prognosis. For instance, if the ovarian cancer had been removed first, the optimal choice of concurrent breast reconstruction after radical mastectomy will have insufficient donor volume from the transverse rectus myocutaneous (TRAM) flap. However, if radical mastectomy with breast reconstruction had been conducted first, the subsequent large intra-abdominal operable lesion site may have impeded primary closure. To overcome this challenge, a synchronous operation was designed by a multidisciplinary oncological and reconstructive team. Herein, we present a rare case using synergic oncological surgery and immediate postmastectomy reconstruction for double primary cancers of the ovary and breast.

## 2. Case presentation

**Figure 1.**
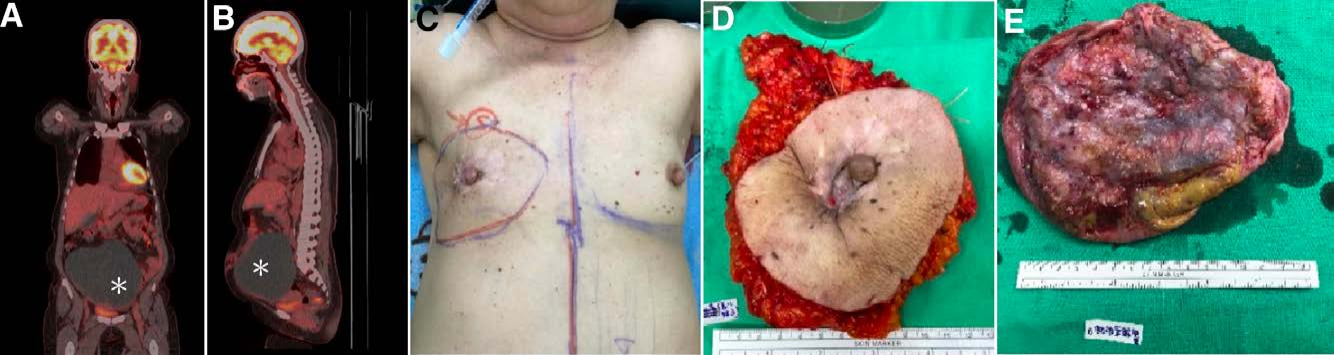
CT images and breast tumor presentation. (A) Coronal and (B) sagittal views of the abdominal mass. (C) Presurgical contouring of breast cancer. (D) Breast cancer lesion. (E) Ovarian tumor. *: right ovarian tumor, size: 17.2 cm × 14.3 cm × 0.3 cm.

A 58-year-old woman presented to the clinic with a right breast lump and ascites. CT images indicated synchronous breast and ovarian cancer with multiple metastases (Fig. 1A,B). Double primary mammary and ovarian cancer was confirmed after a series of evaluations, such as core needle biopsy of the breast tumor. Due to the patient’s special condition, the extended abdominal wall flap seemed to be a good choice for breast reconstruction as a redundant flap with sufficient vascularization. In addition, harvesting the lower abdominal flap exposed the intra-abdominal (gynecological) environment, which simultaneously allowed ovarian cancer removal. Thus, a multidisciplinary team designed a synchronous operation for the removal of double primary cancers and breast reconstruction.

**Figure 2.**
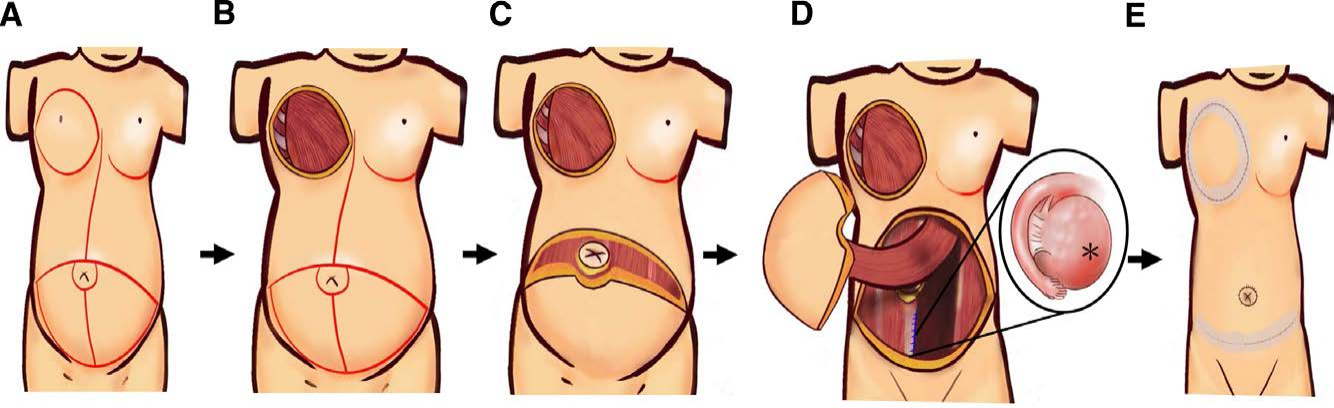
An illustration of synchronous surgery of double cancer and immediate breast reconstruction. (A) Outline of preserved flap volume and mastectomy position. (B) Radical mastectomy completion. (C) TRAM flap harvesting. (D) TRAM flap transfer after bilateral salpingo-oophorectomy. (E) Wound closure. *: right ovarian tumor, size: 17.2 cm × 14.3 cm × 0.3 cm.

The procedure started with right radical mastectomy with axillary lymph node dissection (Fig. 1C,D). Next, plastic surgeons joined to outline the area of the TRAM flap over the lower abdominal area. They made a transverse cesarean section incision to secure the volume of the TRAM flap. The TRAM flap was harvested, and the rectus abdominis was exposed, after which gynecologists joined the surgery for bilateral salpingo-oophorectomy (Fig. 1E). They used a conventional vertical incision over rectus abdominis and created exploratory laparotomy space; the ascitic fluid was drained, and a round 28-cm ovarian tumor with was excised. Next, the plastic surgeons transferred the TRAM flap to the mastectomy site for breast reconstruction (Fig. 2). Umbilical hernia was repaired with a sublay mesh.

**Figure 3.**
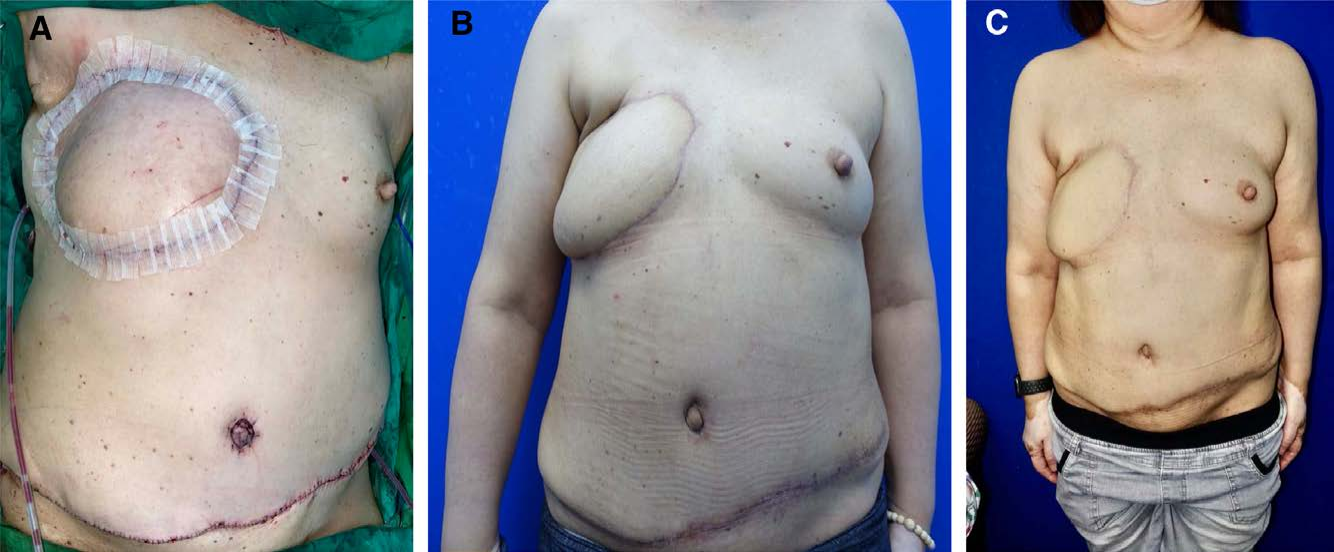
Postoperative follow-ups. (A) Immediately postoperative. (B) Two months after surgery. (C) One year after surgery.

During 1-year follow-up, the patient had exceptional recovery and reported high postoperative satisfaction (Fig. 3). Neither skin contracture nor breast asymmetry was observed during the 1-year follow-up. No postoperative complications were observed, and no abdominal hernia was noted. The ovarian mass was confirmed pathologically as International Federation of Gynecology and Obstetrics (FIGO) stage 1B clear cell carcinoma confined to the right ovary. In addition, because of complete resection of early ovarian cancer, the treatment was successfully downgraded to target therapy for luminal B1 subtype metastatic breast cancer. She could resume her international trading business, with a satisfying quality of life.

## 3. Discussion

Previous studies also mentioned synchronous surgery of ovarian cancer removal and breast reconstruction. Both Khansa et al. and Del Corral et al. suggested that synchronous surgery offered more benefits than sequential surgery such as fewer postoperative complications and shorter stay in hospital (3). Though Tevis et al. suggested that a higher complication rate was found in patients undergoing synchronous surgery (1), our case is unique because a large lower abdominal mass acted as a natural tissue expander, which minimized donor site morbidity while performing TRAM flap harvesting in synchronous surgery. Therefore, on the basis of current standard care for double primary cancer, we formulated three possible surgical plans.

The first approach was prioritizing breast cancer with a TRAM flap over ovarian cancer. After the TRAM flap, the rectus abdominus would be exposed directly below the enlarged abdominal mass. In this situation, ending this surgery and waiting for a second operation by gynecologists seemed nonsensical.

The second approach was prioritizing bilateral salpingo-oophorectomy over mastectomy. However, two choices for breast reconstruction were applying a tissue expander and flap harvesting from other body regions.^[4,5]^ Neither was an ideal option because the tissue expander was the central vertical wound created by ovarian cancer removal, which impeded the integrity of flap volume and because the selection of flaps from other regions was limited.

We, therefore, chose the third approach of synchronous surgery with an aim to solve all the dilemmas in one operation. First, the plastic surgeons preserved the flap volume before ovarian cancer removal, which also minimized the waste of redundant cutaneous volume after ovarian tumor removal. After flap preservation, the rectus abdominus was exposed, allowing the gynecologists to initiate their operation using a principal technique: vertical incision on rectus abdominus. Thus, the flap was preserved without affecting salpingo-oophorectomy. Since the required flap volume was expanded naturally by ovarian tumor outgrowth, the TRAM flap was deemed well-vascularized and ready for instant transfer. Because of its sufficient vascularization, all four zones of the TRAM flap could be utilized to completely restore the volume of the breast. Thus, a successful mastectomy followed by immediate breast reconstruction could be predicted. After complete tumor removal, plastic surgeons resumed flap transfer and breast reconstruction and completed the abdominal wound closure.

In conclusion, this case is unique because the synchronous operation design not only optimally made use of the redundant abdominal skin volume but also minimized donor site morbidity. No skin graft or flap transfer was needed after TRAM flap harvesting. Moreover, complete removal of ovarian cancer, reduction of breast cancer, excellent breast reconstruction, abdominal hernia repair, and body shape recovery were also achieved at the same time. The patient had an excellent prognosis and good quality of life. This approach can be a novel treatment option for selected cases of double primary cancers instead of two operations, thereby reducing morbidity and costs.

## Author contributions

CHH contributed to the acquisition of patient information and manuscript writing. YRK and YCC participated in the diagnosis and treatment of the patient. SLS revised the manuscript and all authors read and approved the final manuscript. CHH, YRK, and YCC investigated the study. SLS supervised the study. CHH wrote the original draft. YCC and YRK wrote reviewed and edited the manuscript.
